# Intestinal rearrangement of biliopancreatic limbs, alimentary limbs, and common limbs in obese type 2 diabetic mice after duodenal jejunal bypass surgery

**DOI:** 10.3389/fendo.2024.1456885

**Published:** 2025-01-08

**Authors:** Heng Li, Jipei He, Jie Hou, Chengjun He, Xiaojiang Dai, Zhigao Song, Qing Liu, Zixin Wang, Hongyan Huang, Yunfa Ding, Tengfei Qi, Hongbin Zhang, Liangping Wu

**Affiliations:** ^1^ Department of Metabolic Surgery, Jinshazhou Hospital of Guangzhou University of Chinese Medicine, Guangzhou, China; ^2^ Department of Endocrinology and Metabolism, Third Affiliated Hospital of Sun Yat-Sen University, Guangzhou, China; ^3^ Department of Basic Medical Research, General Hospital of Southern Theater Command of People's Liberation Army (PLA), Guangzhou, China; ^4^ Department of Cardiovascular Surgery, Zhujiang Hospital of Southern Medical University, Guangzhou, China; ^5^ Zhongshan Institute for Drug Discovery, Shanghai Institute of Materia Medica, Chinese Academy of Sciences, Guangzhou, China; ^6^ School of Laboratory Medicine and Biotechnology, Southern Medical University, Guangzhou, China; ^7^ Guangzhou Hualiang Qingying Biotechnology Co. Ltd, Guangzhou, China

**Keywords:** duodenal jejunal bypass, type 2 diabetes mellitus, gut microbiota, gut barrier, metabonomics, bariatric surgery

## Abstract

Bariatric surgery is an effective treatment for type 2 Diabetes Mellitus (T2DM), yet the precise mechanisms underlying its effectiveness remain incompletely understood. While previous research has emphasized the role of rearrangement of the gastrointestinal anatomy, gaps persist regarding the specific impact on the gut microbiota and barriers within the biliopancreatic, alimentary, and common limbs. This study aimed to investigate the effects of duodenal-jejunal bypass (DJB) surgery on obese T2DM mice. We performed DJB and SHAM surgery in obese T2DM mice to investigate changes in the gut microbiota and barrier across different intestinal limbs. The effects on serum metabolism and potential associations with T2DM improvement were also investigated. Following DJB surgery, there was an increased abundance of commensals across various limbs. Additionally, the surgery improved intestinal permeability and inflammation in the alimentary and common limbs, while reducing inflammation in the biliopancreatic limbs. Furthermore, DJB surgery also improved T2DM by increasing L-glutamine, short-chain fatty acids, and bile acids and decreasing branched-chain amino acids. This study underscores the role of intestinal rearrangement in reshaping gut microbiota composition and enhancing gut barrier function, thereby contributing to the amelioration of T2DM following bariatric surgery, and providing new insights for further research on bariatric surgery.

## 1 Introduction

Bariatric surgery is increasingly performed worldwide to treat morbid obesity and is also known as metabolic surgery due to its beneficial metabolic effects, especially with respect to improvement in type 2 Diabetes Mellitus (T2DM) (1). In T2DM, relative insulin deficiency resulting from β-cell dysfunction is a key factor contributing to disease development, often in conjunction with insulin resistance (2). With the evolution of metabolic surgery, it has emerged as a viable long-term intervention for treating diabetes (3).

Roux-en-Y gastric bypass (RYGB) is a highly effective treatment for severe obesity and type 2 diabetes. By inducing alterations in the anatomical structure of the gastrointestinal tract, RYGB modifies the gut microbiota and diminishes systemic endotoxemia (4, 5). Anatomical rearrangement of the gastrointestinal tract likely alters the composition of the luminal milieu, consequently influencing downstream signaling pathways that regulate host energy balance and metabolism (1). Previous studies have highlighted the importance of jejunal and duodenal nutrient sensing in blood glucose homeostasis (6) with studies indicating that nutrient infusion bypassing the duodenum enhances insulin sensitivity (7). Rubino et al. have identified the proximal jejunum’s involvement in the pathogenesis of T2DM (8). However, the mechanisms underlying these effects are not fully understood. To address these knowledge gaps, we adopted the recently developed duodenojejunal bypass (DJB) mouse model. DJB is a metabolic procedure involving the exclusion of nutrients from the duodenum and proximal jejunum, followed by jejunal Roux-en-Y reconstruction and early nutrient delivery to the distal small bowel (9). Intestinal remodeling following DJB surgery preserves the physiological structure of the stomach and provides an avenue for investigating the mechanism by which the proximal jejunum improves metabolism.

Combining 16S rRNA gene sequencing with metabolomics is considered a reliable method for analyzing structural changes in the gut microbiota and the metabolic profiles of the gut microbiota and the host (10). The gut microbiota is crucial for many biological functions in the body, including intestinal development, barrier integrity and function, metabolism, and the immune system (11, 12). In healthy individuals, the intestinal barrier consists of a cohesive layer of epithelial cells connected by tight junctions (TJ) (13). However, metabolites originating from the gut microbiota can enter the circulatory system, breaching the intestinal barrier to influence distal organs and potentially impact the progression of T2D (10). Increasing evidence has shown compositional differences in the gut microbiota and their metabolic characteristics, as well as the relationship between the intestinal microbiota and metabolism in T2D patients and healthy individuals (14). In this study, we used 16S rRNA gene sequencing and metabolomics to analyze the changes in the proximal gut microbiota and serum metabolism, aiming to elucidate their contributions to the DJB effect. The proximal jejunum was surgically treated in three separate sections, each of which may contribute to different local responses of the gut microbiota.

## 2 Materials and methods

### 2.1 Animals

Twenty 8-week-old male C57BL/6J mice were purchased from China Yaokang Cavens Laboratory Animal Center, housed in a specific pathogen-free (SPF) laboratory, and subjected to a light/dark cycle for 12 hours. Temperature was maintained at 22 ± 2℃ and humidity 55−65%. For six weeks, the mice were provided water and a diet containing 60% of calories from fat. To induce diabetes, intraperitoneal streptozotocin (40 mg/kg) was administered at week 7 for 5 consecutive days. Among the twenty mice, fourteen mice were screened for random blood glucose > 16.7 mmol/L and were randomly assigned to DJB (n=9) and SHAM (n=5) surgery groups. Six mice were excluded from the experiment due to substandard blood glucose. Nine mice underwent DJB surgery, five mice survived.

### 2.2 Surgical procedure

**Figure 1:**
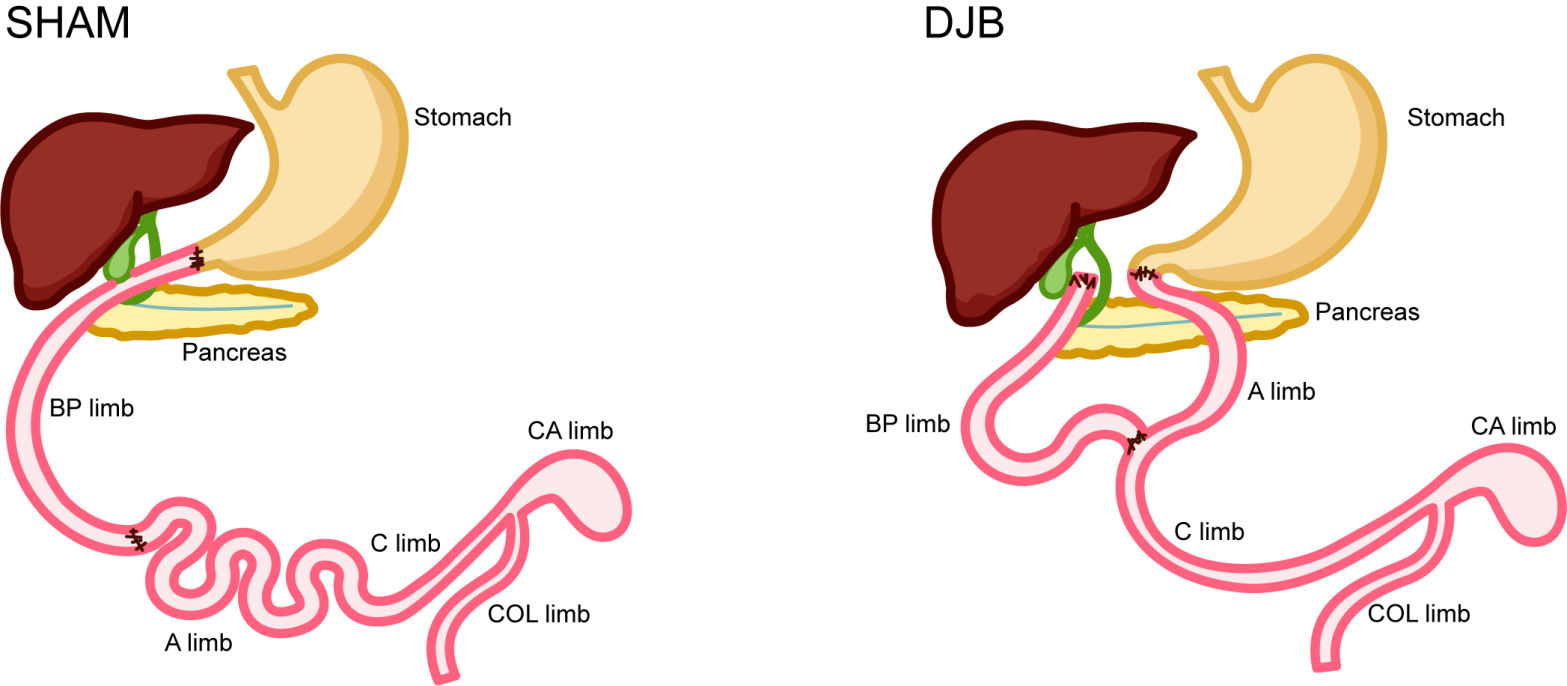
Operations and intestinal sampling locations. SHAM operation. Duodenal jejunal bypass (DJB) operation. BP limb, biliopancreatic limb; A limb, alimentary limb; C limb, common limb; CA limb, caecum limb; COL limb, colon limb.

The DJB surgical procedures were performed as described in the **Supplementary Material**. For SHAM surgery, gastrointestinal transection and re-anastomosis are performed at a similar site as DJB (**Figure 1**).

### 2.3 Measurement of food intake, body weight, and blood glucose

The mice were allowed to recover for 1 week after surgery. Mice were individually housed in cages, and food intake was measured by weighing the amount of solid food before and after 24 hours. The body weights and random blood glucose levels of the mice were recorded before and 8 weeks after surgery. The mice were allowed to fast for 8 hours before the oral glucose tolerance test (OGTT) experiment in the 8th week after surgery, wherein an oral bolus of 20% D-glucose (2 g/kg) was administered to the mice, and blood glucose levels were measured at 0, 15, 30, 60, 90, 120, 150, and 180 min after gavage. Blood glucose levels were measured in blood collected from the tail vein using a handheld AccuChek Performa Glucometer (Roche).

### 2.4 Sample collection

At 8 weeks post-surgery, the mice were fasted overnight, and then euthanized to collect blood samples, which were stored at room temperature for 2-4 h, and then centrifuged at 3000 rpm for 10 min to extract the sera. Carefully collected intestinal contents and intestinal tissues of the biliopancreatic limb (BP limb), alimentary limb (A limb), common limb (C limb), caecum limb (CA limb), and colon limb (COL limb) were promptly frozen in liquid nitrogen and stored at −80°C. Representative sections of the intestinal segments were also collected from the SHAM animals for comparison. Histological analysis was performed on intestinal tissues.

### 2.5 RNA extraction and quantitative real time–ploymerase chain reaction (qRT-PCR)

Total RNA was extracted from intestinal tissues using an EZ-press RNA Purification Kit (EZ Bioscience, Shanghai, China) and reverse transcribed into cDNA using an EZscript Reverse Transcription Mixture (EZ Bioscience, Shanghai, China) according to the manufacturer’s protocol. qRT-PCR was performed using the SYBR Green Master Mix (EZBioscience, Shanghai, China). The results were normalized as relative values of GAPDH mRNA, and the data were analyzed according to 2^-ΔΔCT^. The primer sequences are listed in **Supplementary Table 1**.

### 2.6 Hematoxylin-eosin staining and immunohistochemistry

A hematoxylin and eosin (HE) staining kit (Beyotime Biotechnology, Shanghai, China) was used to stain the mouse intestinal tracts. The tissues were fixed with 4% paraformaldehyde for 24 hours and dehydrated with ethanol. The tissues were cleaned with xylene, embedded in paraffin, cut into 5 μm slices, dewaxed and dehydrated, and stained with hematoxylin for 5 min and eosin for 3 min. Morphology of the small intestine was observed under a microscope, and immunohistochemical (IHC) analysis was performed as previously described (15).

### 2.7 Immunofluorescence

Paraffin sections (4 μm thick) were dewaxed, rehydrated, treated with ethylenediamine tetraacetic acid antigen recovery solution (Beyotime, China), and blocked with 5% goat serum at 37℃ for 1 h. Subsequently, the sections were incubated with a primary antibody (anti-ZO-1and anti-Claudin-5; Abcam) at 4°C overnight, followed by Alexa Fluor 488 goat-anti-rabbit immunoglobulin G (Cell Signaling Technology) and Alexa Fluor 555 goat-anti-mouse IgG (Cell Signaling Technology) for 1 h at 37°C. Finally, the slices were cleaned and reverse-stained with DAPI (Cell Signaling Technology) using a 20× lens to obtain 3–5 images per slice.

### 2.8 Enzyme-linked immunosorbent assay (ELISA)

Sera were stored at −80°C until analysis. Serum cytokines interleukin-1 β (IL-1β), interleukin-6 (IL-6), tumor necrosis factor-α (TNF-α), and glucagon-like peptide 1 (GLP-1) were measured using ELISA kits according to the manufacturer’s instructions (Ray Biotech, USA).

### 2.9 Metabolomic analysis

Based on previous studies, we performed a metabolomic analysis of serum content using liquid chromatography-mass spectrometry (LC-MS/MS) (16).

### 2.10 16S rRNA gene sequencing analysis

Absolute quantification of 16S rRNA amplicon sequencing was performed by Majorbio Bio-Pharm Technology Co., Ltd. (Shanghai, China). Total microbial genomic DNA was extracted using E.Z.N.A.^®^ soil DNA Kit (Omega Bio-Tek, Norcross, GA, U.S.) according to the manufacturer’s instructions. The V3-V4 hypervariable portions of the bacterium 16S rRNA gene were amplified using a thermocycler PCR system (GeneAmp 9700, ABI, USA) with primers 338F (5′-ACTCCTACGGGAGGCAGCAG-3′) and 806R (5′-GGACTACHVGGGTWTCTAAT-3′). Purified amplicons were pooled in equimolar amounts and paired-end sequenced on an Illumina PE250 platform (Illumina, San Diego, CA, USA) according to the standard protocols of Majorbio Bio-Pharm Technology Co. Ltd. (Shanghai, China). Raw sequencing reads were deposited in the NCBI under the BioProject ID PRJNA1087303.

### 2.11 Statistical analysis

Data were analyzed using GraphPad Prism software and expressed as the mean ± standard deviation (SD). The area under the curve was calculated using the trapezoidal rule. All images were analyzed using Image-Pro Plus 6.0 (Media Cybernetics, USA). An unpaired Student’s *t*-test was used to determine the significance of the intergroup differences, and values of *P*< 0.05 were considered significant. The Majorbio I-Sanger Cloud Platform (www.i-sanger.com) was utilized to examine the 16S rRNA gene sequencing data.

## 3 Results

### 3.1 Effects of DJB on obese T2D mice model

**Figure 2:**
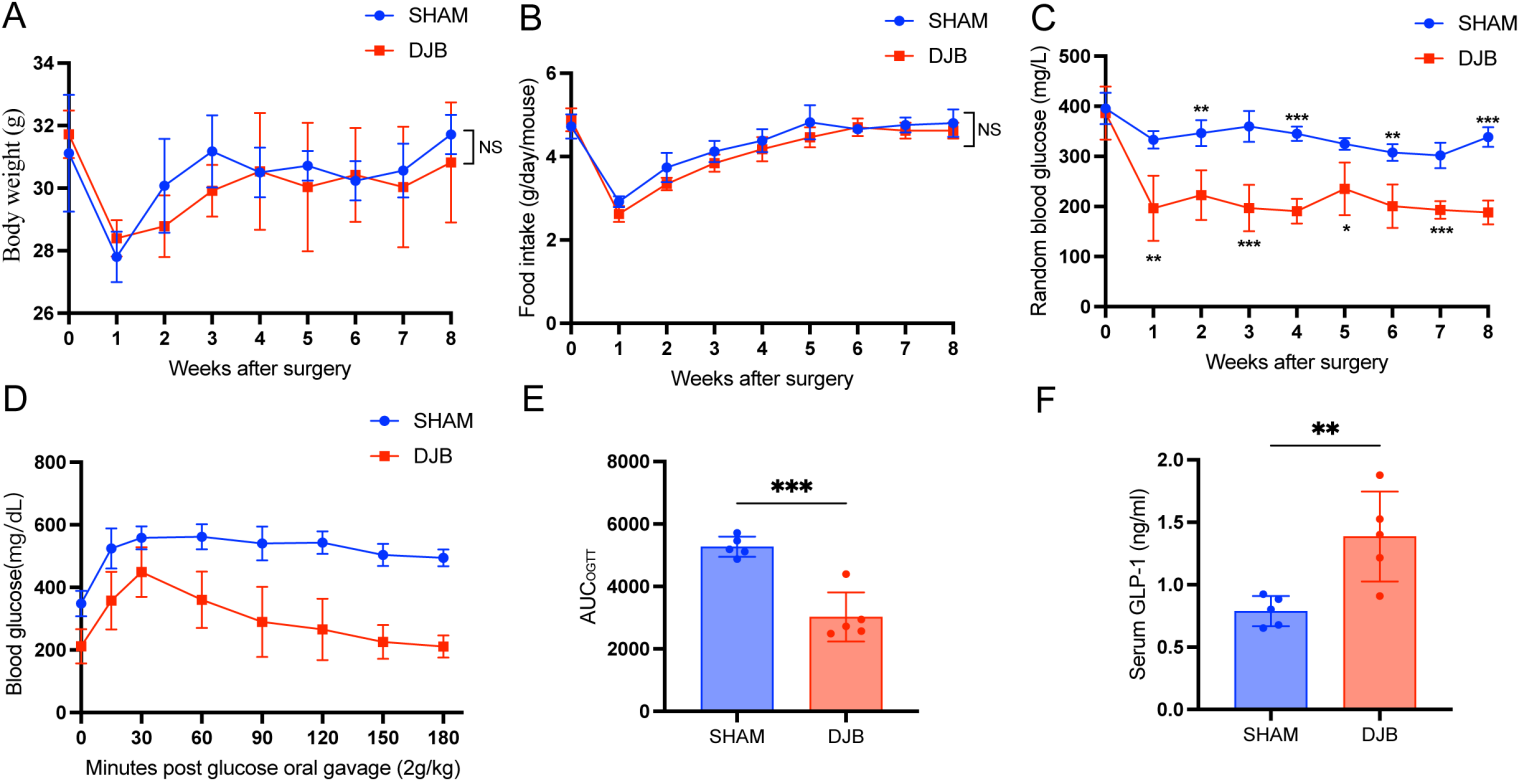
DJB improved glucose metabolism in mice with T2D. **(A)** Body weight. **(B)** Food intake. **(C)** Random blood glucose. **(D, E)** Oral glucose tolerance test (OGTT) and area under the curve (AUC). **(F)** The quantitative levels of GLP-1 in serum between DJB and SHAM groups detected by ELISA. The data are shown as mean ± SD, n=5. Statistical analyses were performed by a two-tailed, unpaired Student’s t-test. ^*^ *P* < 0.05, ^**^ *P* < 0.01, ^***^ *P* < 0.001. OGTT, oral glucose tolerance test; AUC_OGTT_, the area under the OGTT curve; GLP-1, glucagon-like peptide 1; NS, no significance.

Within 8 weeks of DJB, no significant differences in body weight were observed (**Figure 2A**), nor in food intake (**Figure 2B**) in both DJB and SHAM animals; however, a notable weight control effect was evident after DJB surgery. As anticipated, significant reductions were observed in blood glucose levels and the area under the OGTT curve indicating improved glucose tolerance (**Figures 2C-E**). GLP-1 levels were also significantly elevated (**Figure 2F**) in DJB animals than in SHAM animals. These findings underscore significant improvements in glucose tolerance and insulin sensitivity following DJB surgery.

### 3.2 DJB modulated gut microbiota composition in obese T2D mice

**Figure 3:**
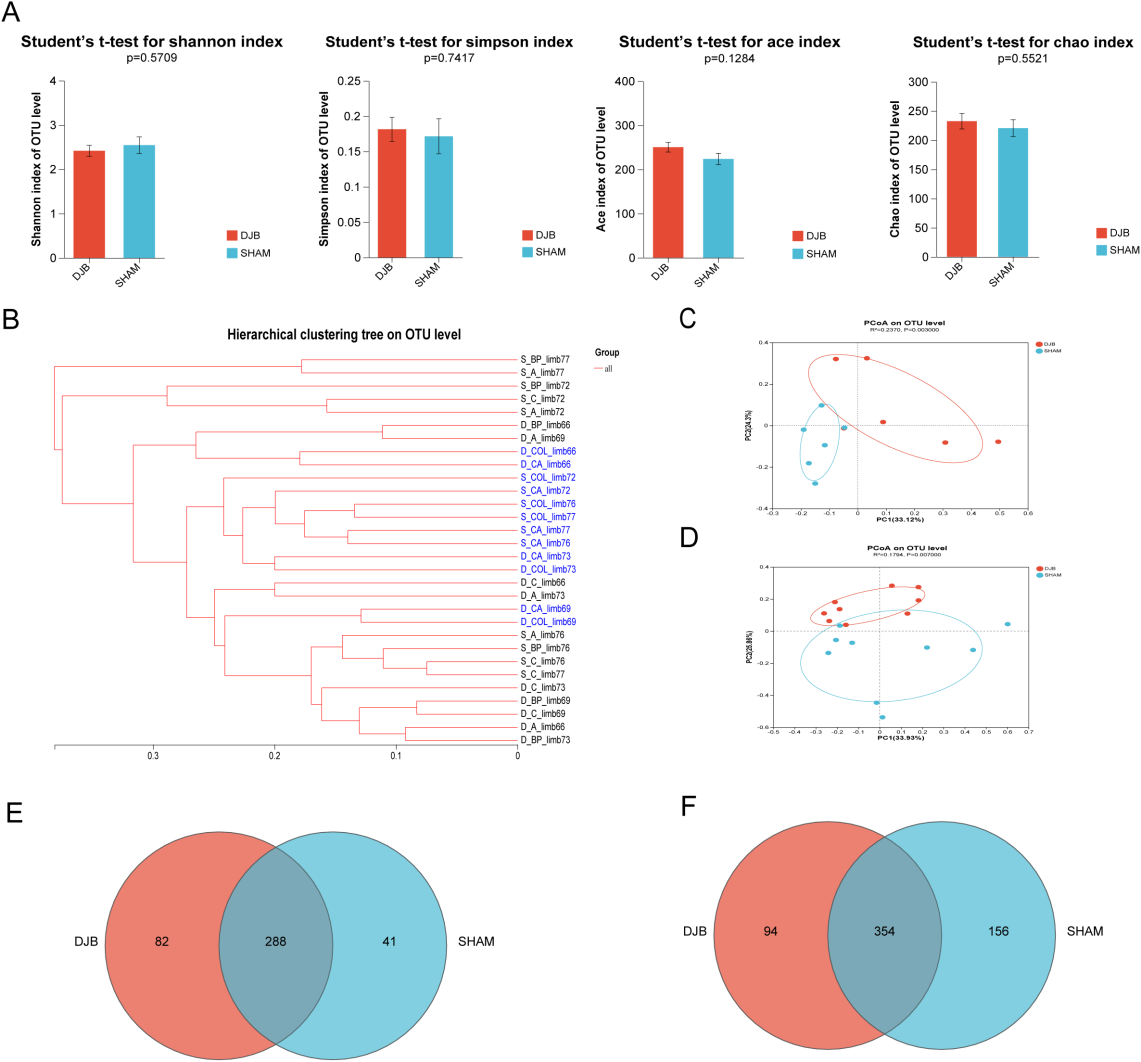
Overall effects of DJB on gut microbiota. **(A)** Alpha-diversity indexes including Shannon, Simpson, Ace, and Chao. **(B-D)** Beta-diversity was presented as a hierarchical clustering tree and PCoA at the OTU level. **(C)** PCoA shows the Bray-Curtis distance in the CA and COL limbs between DJB and SHAM groups, n=6. **(D)** PCoA shows the Bray-Curtis distance in the BP, A, and C limbs between DJB and SHAM groups. Each point represents each sample, n=9. **(E)** Venn diagram of common OTUs in the CA and COL limbs, n=6. **(F)** Venn diagram of common OTUs in the BP, A, and C limbs, n=9. The data are shown as mean ± SD. Statistical analyses were performed by a two-tailed, unpaired Student’s *t*-test. BP limb, biliopancreatic limb; A limb, alimentary limb; C limb, common limb; CA limb, caecum limb; COL limb, colon limb.

In this study, we analyzed the 16S ribosomal RNA (rRNA) sequences of proximal (BP, A, and C limbs) and distal (CA and COL limbs) gut samples. Bioinformatic analysis of the resulting sequences for operational taxonomic units (OTUs) at 97% similarity revealed no differences in the alpha-diversity indices (Shannon, Simpson, Ace, and Chao) of the gut microbiota in the DJB and SHAM groups (*P* > 0.05, **Figure 3A**). Beta diversity, assessed using a hierarchical clustering tree, showed that the gut microbiota was distinctly clustered among the proximal and distal samples at the OTU level. (**Figure 3B**). Principal coordinate analysis (PCoA) was analyzed at the OTU level in the distal region (*P* < 0.05; **Figure 3C**) and proximal *(P* < 0.05, **Figure 3D**) samples, suggesting a significant difference in the intestinal microbiome composition between the DJB and SHAM groups. Furthermore, 411 and 604 OTUs were found in the distal (**Figure 3E**) and proximal regions (**Figure 3F**), as shown in the Venn diagram. Overall, our results indicated that the proximal and distal gut microbiota play different roles after DJB surgery.

### 3.3 DJB caused different segmental changes in the distal and proximal gut microbiota

**Figure 4:**
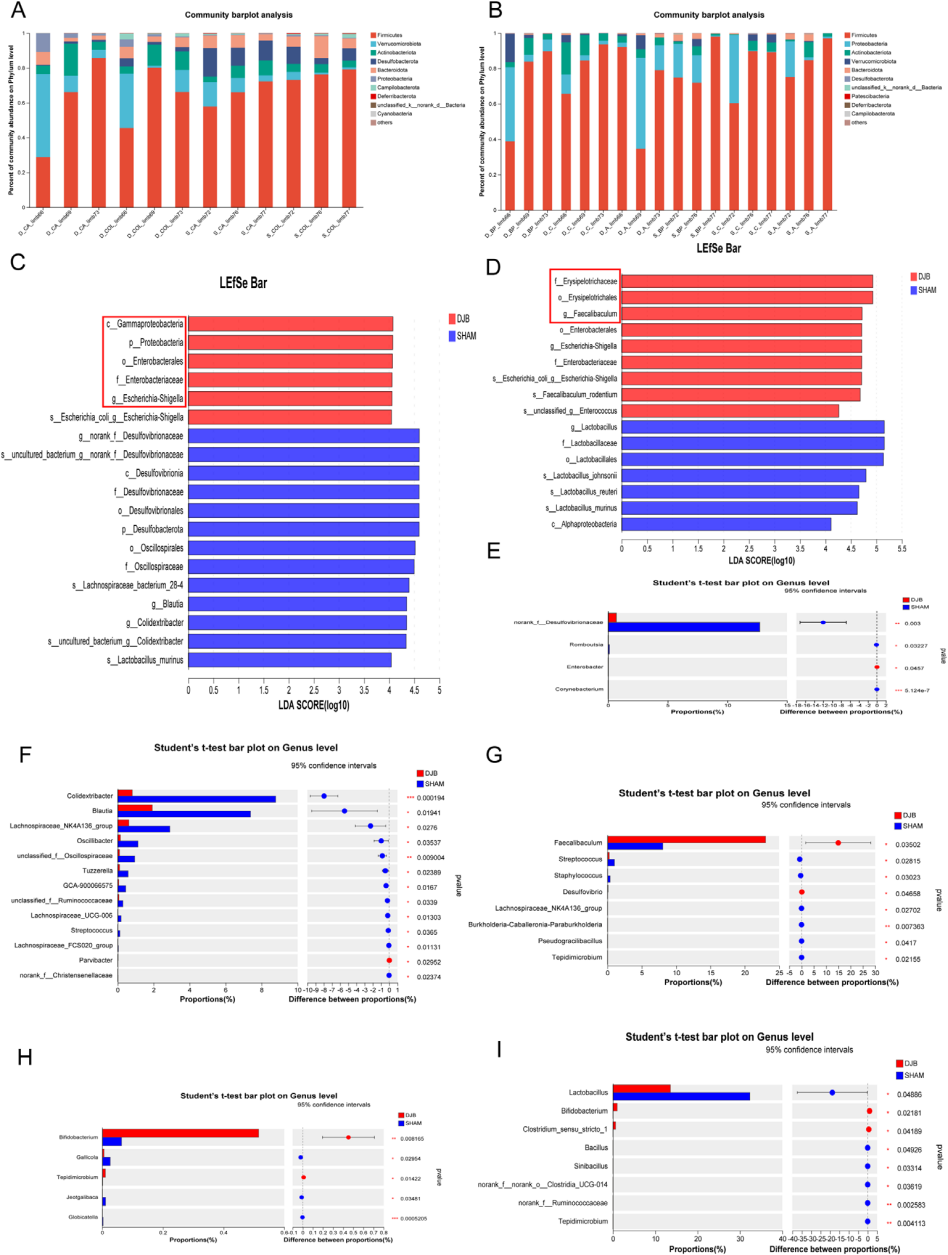
DJB caused different segmental changes in the distal and proximal intestinal limbs. **(A)** The relative abundance of gut microbiota between the CA and COL limbs at the phylum level. **(B)** The relative abundance of gut microbiota between the BP, A, and C limbs at the phylum level. **(C)** Linear discriminant analysis (LDA) effect size (LEfSe) analysis of microbiota composition in the CA and COL limbs between DJB and SHAM groups (LDA > 4, n=6). **(D)** Linear discriminant analysis (LDA) effect size (LEfSe) analysis of microbiota composition in the BP, A, and C limbs between DJB and SHAM groups (LDA > 4, n=9). **(E)** Bacteria with significant changes in the relative abundance in limb CA at the genus level, n=3. **(F)** Bacteria with significant changes in the relative abundance in limb COL at the genus level, n=3. **(G)** Bacteria with significant changes in the relative abundance in limb BP at the genus level, n=3. **(H)** Bacteria with significant changes in the relative abundance in limb A at the genus level, n=3. **(I)** Bacteria with significant changes in the relative abundance in limb C at the genus level, n=3. The data are shown as mean ± SD. Statistical analyses were performed by a two-tailed, unpaired Student’s *t*-test. ^*^ *P* < 0.05, ^**^ *P* < 0.01, ^***^ *P* < 0.001. LDA, Linear discriminant analysis; BP limb, biliopancreatic limb; A limb, alimentary limb; C limb, common limb; CA limb, caecum limb; COL limb, colon limb.

Next, we investigated the effects of DJB surgery on the gut microbiota in the distal and proximal gut. Analysis of the gut microbial composition at the phylum level revealed no differences between the distal and proximal limbs (**Figures 4A, B**). However, the dominant microorganisms in the groups were explored using the linear discriminant analysis effect size (LEfSe) analysis (Linear discriminant analysis, LDA > 4, *P* < 0.05). In the CA and COL limbs, LEfSe analysis confirmed the enrichment of Proteobacteria, Gammaproteobacteria, Enterobacterales, and Enterobacteriaceae (**Figure 4C**). These changes are similar to those observed in RYGB animals (17). Conversely, the Faecalibaculum and Enterobacterales were enriched in DJB in the BP, A, and C limbs (**Figure 4D**). At the genus level, the relative abundance of norank_f_Desulfovibrionaceae in the CA limbs (*P* < 0.05, **Figure 4E**) and Colidextribacter, Blautia, and Lachnospiraceae_NK4A136_group in the COL limbs (*P* < 0.05, **Figure 4F**) significantly decreased after DJB (**Supplementary Table 2**). In addition, DJB mice exhibited a higher abundance of Faecalibaculum in the BP limbs (*P* < 0.05, **Figure 4G**) and Bifidobacterium in limb A (*P* < 0.05, **Figure 4H**). DJB surgery significantly decreased the abundance of Lactobacillus in the C limbs (*P* < 0.05; **Figure 4I** and **Supplementary Table 3**).

According to our findings, the changes in distal gut microbes were consistent with previous studies on bariatric surgery (17). However, most studies have focused on distal gut microbiota, such as fecal microbiota, neglecting the equally important proximal intestinal gut microbiota. In the analysis of bacterial differences, we found that DJB surgery increased the abundance of beneficial bacteria in the BP, A, and C limbs compared to the distal gut. Therefore, we believe that intestinal rearrangement after DJB surgery leads to the dominance of the proximal small intestine in improving glucose metabolism.

### 3.4 DJB improved the gut barrier of biliopancreatic limbs, alimentary limbs, and common limbs

**Figure 5:**
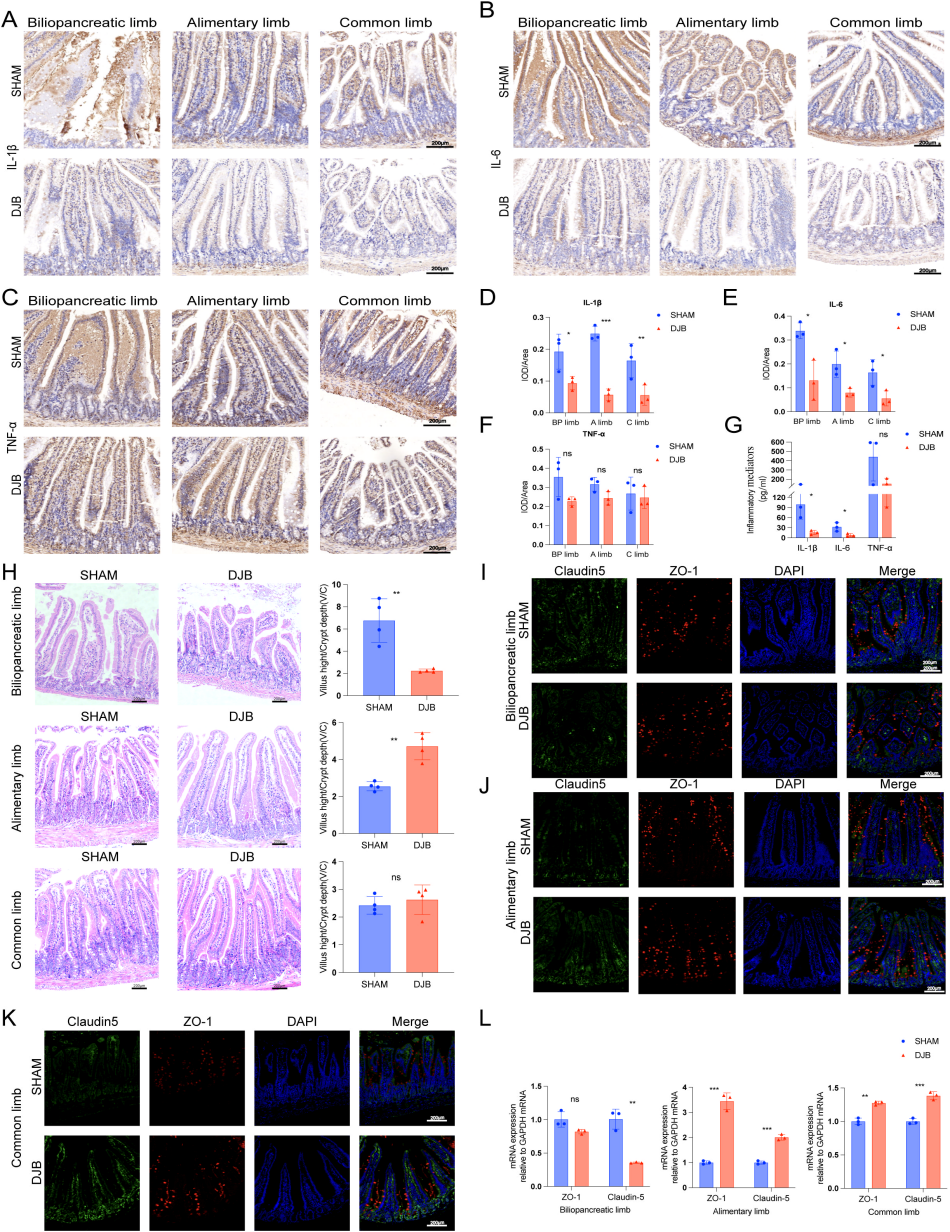
DJB improved the gut barrier in BP limbs, A limbs, and C limbs. Representative micrographs of Immunohistochemical staining **(A)** and quantitative data **(D)** of IL-1β in intestinal tissue sections of each group of mice, n=3. Representative micrographs of Immunohistochemical staining **(B)** and quantitative data **(E)** of IL-6 in intestinal tissue sections of each group of mice, n=3. Representative micrographs of Immunohistochemical staining **(C)** and quantitative data **(F)** of TNF-α in intestinal tissue sections of each group of mice, n=3. **(G)** The quantitative levels of IL-1β, IL-6, and TNF-α in the serum of each group of mice detected by ELISA, n=3. **(H)** Representative micrographs of HE staining and quantitative analysis of V/C in the BP, A, and C limbs, n=4. **(I)** Double-immunofluorescence staining analysis of Claudin-5 and ZO-1 in the biliopancreatic limbs. **(J)** Double-immunofluorescence staining analysis of Claudin-5 and ZO-1 in the alimentary limbs. **(K)** Double-immunofluorescence staining analysis of Claudin-5 and ZO-1 in the common limbs. **(L)** The mRNA levels of ZO-1 and Claudin-5 were detected by qRT-PCR in intestinal tissue sections of each group of mice, n=3. The data are shown as mean ± SD. Statistical analyses were performed by two-tailed, unpaired Student’s *t*-test. ^*^ *P* < 0.05, ^**^ *P* < 0.01, ^***^ *P* < 0.001. Scales bars = 200 μm. ZO-1, zonula occludens 1; IL-1β, interleukin-1β; TNF-α, tumor necrosis factor-α; IL-6, interleukin-6; BP limb, biliopancreatic limb; A limb, alimentary limb; C limb, common limb; ns, no significance.

To determine whether the proximal gut microbiota affects the gut barrier, we examined inflammation and permeability in different segments of the proximal jejunum after DJB. The IHC results showed that the expression of IL-1β (*P* < 0.05, **Figures 5A, D**) and IL-6 (*P* < 0.05; **Figures 5B, E**) in BP, A, and C limbs were significantly decreased after DJB, as well as the expression of TNF-α in the three intestinal segments (*P* > 0.05, **Figures 5C, F**) compared to the SHAM group. In addition, we detected a decreased serum level of IL-1β, IL-6, and TNF-α in DJB groups (**Figure 5G**). Thus, DJB surgery can be inferred to have reduced inflammation in obese T2D mice. Meanwhile, DJB surgery decreased the villus height/crypt depth (V/C) of the BP limbs (**Figure 5H**). Compared with the SHAM group, the (V/C) of the A limbs increased significantly in the DJB group, and there was no significant change in the C limbs. After DJB, mice exhibit significant adaptive intestinal changes (18).

To further elucidate the effect of DJB on the mucosal barrier of the proximal small intestine, immunofluorescence and qRT-PCR were used to detect the expression of zonula occludens 1 (ZO-1) and claudin-5, respectively. Immunofluorescence analysis showed that ZO-1 and claudin-5 were strongly expressed in the intestinal surface epithelial cells of A (**Figure 5J**) and C limbs (**Figure 5K**) after DJB surgery, showing characteristic lateral membrane staining. After DJB, staining was concentrated at the tips of the villi and cells in the crypt. However, there were no significant changes in the BP limbs (**Figure 5I**). Similar changes were also found by qRT-PCR, with increased expression levels of ZO-1 and claudin-5 mRNA in the A and C limbs, and decreased expression levels in the BP limbs (**Figure 5L**). These findings suggest that DJB maintains intestinal epithelial homeostasis by regulating TJ protein distribution and expression.

### 3.5 Changes in the overall status of the metabolome after DJB surgery

**Figure 6:**
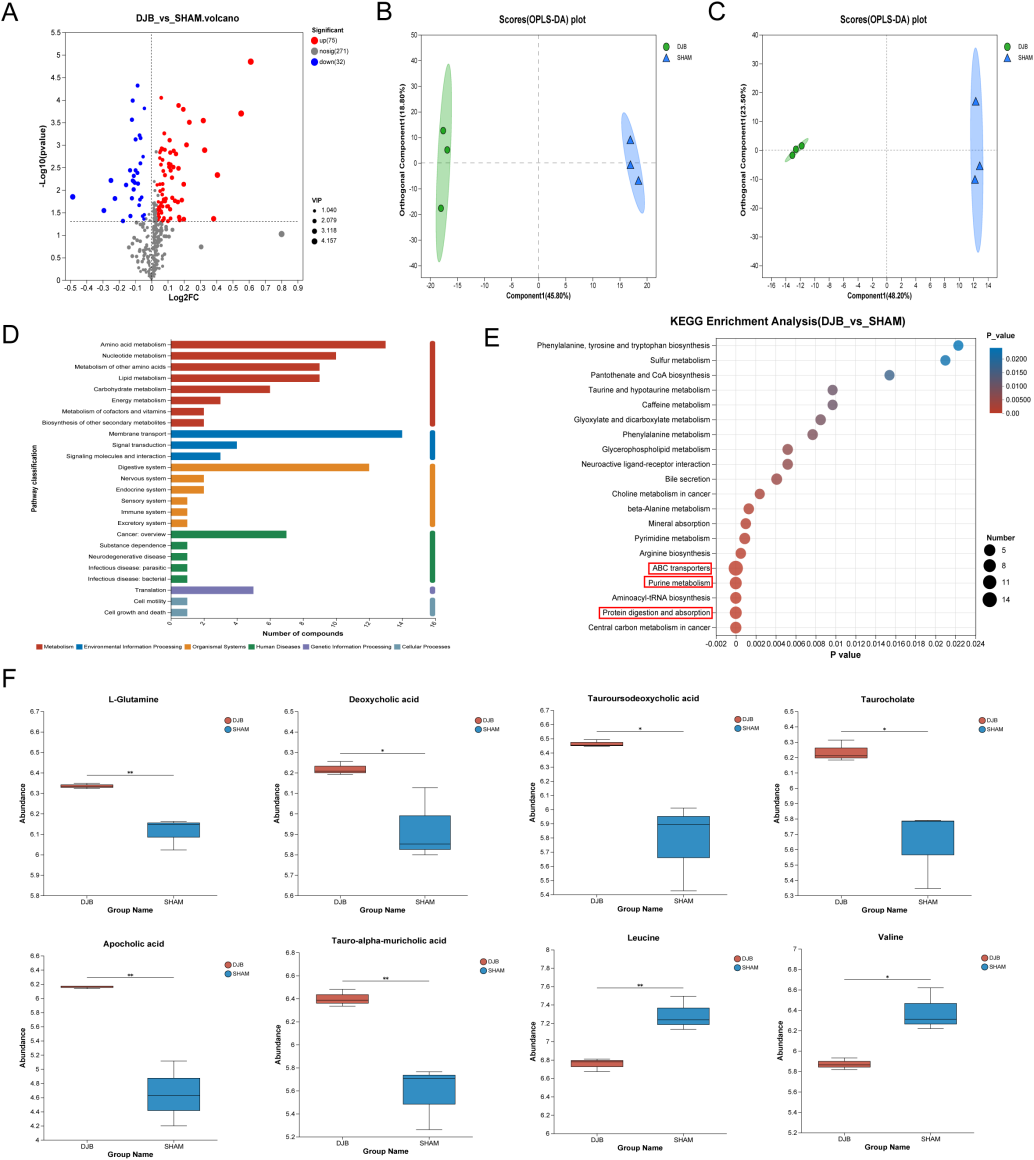
LC–MS-based metabolomics analysis of the serum samples from DJB and SHAM groups. **(A)** Volcano map of metabolites between DJB and SHAM groups. Red dots represent significantly upregulated metabolites, and blue dots represent significantly downregulated metabolites, and gray dots represent non-significant differential metabolites. The differential metabolites were identified as VIP > 1 and P < 0.05. **(B)** Orthogonal partial least squares discriminant analysis (OPLS-DA) map of a positive ion. **(C)** OPLS-DA map of negative ion. **(D)** KEGG pathways on level 1 and level 2 are related to differential metabolites. The ordinate is the name of pathway level 2, and the abscissa is the number of metabolites related to the pathway. Different colors represent different pathways on level 1. **(E)** Bubble diagram showing the enriched KEGG pathways. The ordinate is the name of Pathway 3. The size of the bubbles in the figure represents how much of the pathway is enriched into the metabolic compound. Generally, a p value less than 0.05 is considered a significant enrichment item. **(F)** Comparison of the relative abundance of L-Glutamine, Deoxycholic acid, Tauroursodeoxycholic acid, Taurocholate, Apocholic acid, Tauro-alpha-muricholic acid, Leucine, and Valine between DJB and SHAM groups, n=3. The data are shown as mean ± SD. ^*^ *P* < 0.05, ^**^ *P* < 0.01.

To identify the potential metabolic signals that could lead to the loss of blood glucose levels, we examined the effects of DJB surgery on the serum metabolism profile of mice. A total of 378 metabolites were identified. In total, 107 differential metabolites were detected (**Supplementary Table 4**), of which 75 were upregulated and 32 were downregulated (**Figure 6A**). Orthogonal partial least squares discriminant analysis (OPLS-DA) showed (**Figures 6B, C**) that DJB surgery significantly altered the metabolic profile of diabetic mice.

In addition, the Kyoto Encyclopedia of Genes and Genomes (KEGG) analysis of the 107 differential metabolites showed significant enrichment of pathways related to membrane transport, amino acid metabolism, and the digestive system (**Figure 6D**). To further identify the pathways affected by DJB, we performed an enrichment analysis of the KEGG pathway for important metabolites with known KEGG IDs among which the top 20 pathways are shown in **Figure 6E** (*P* < 0.05). Changes in metabolic pathways were mainly related to ABC transporters, purine metabolism, and protein digestion and absorption pathways. Interestingly, L-Glutamine is involved in these metabolic pathways (**Figure 6F** and **Supplementary Table 5**). In addition, we found that bile acids (deoxycholic acid, tauroursodeoxycholic acid, taurocholate, apolicholic acid, and tauro-alpha-muricholic acid), branched-chain amino acids (Leucine and Valine) (**Figure 6F**), and three short-chain fatty acids (SCFAs) (**Supplementary Table 6**) were elevated after DJB.

### 3.6 Correlation analysis between metabolism and gut microbiota

**Figure 7:**
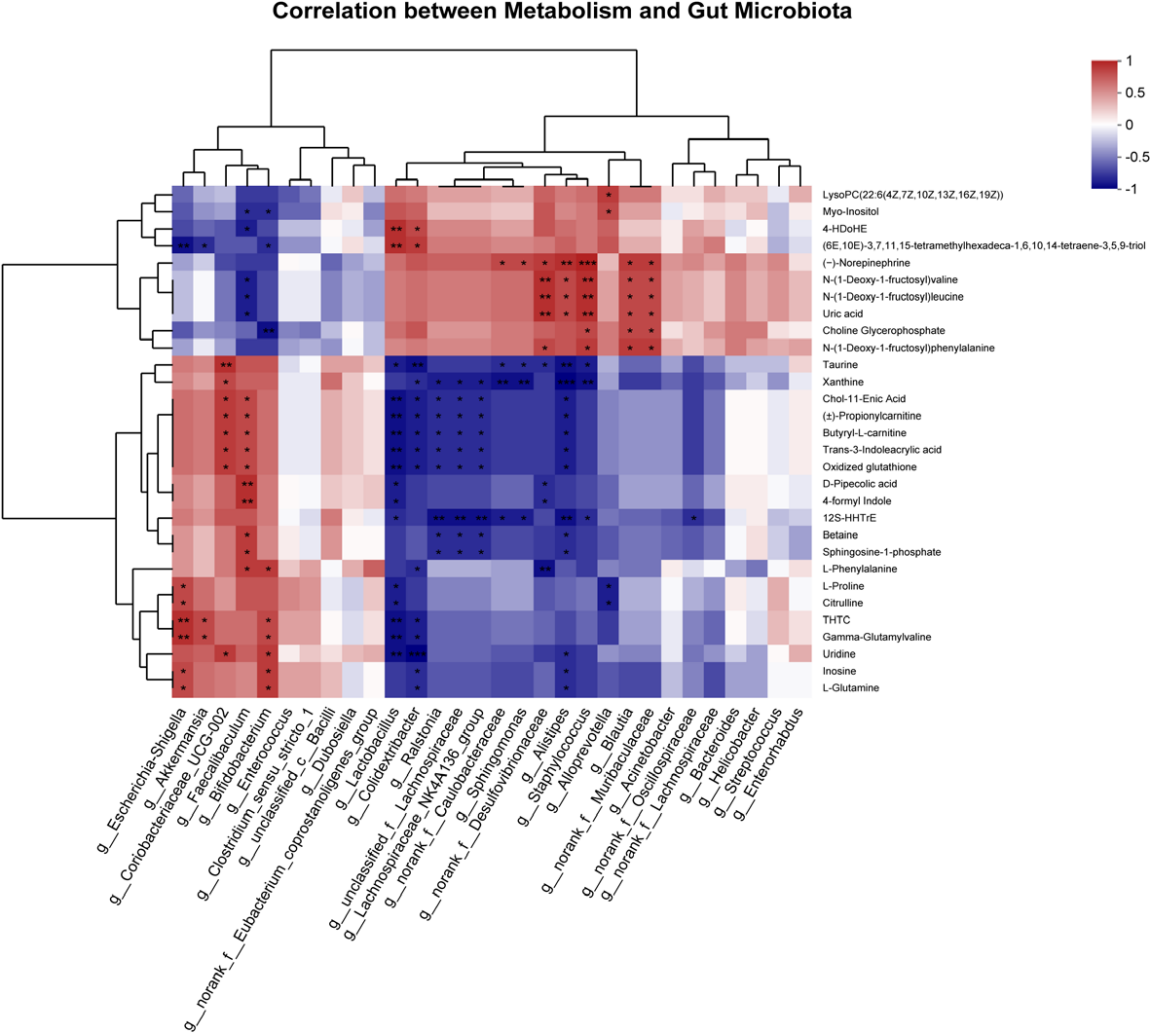
Spearman correlation analysis of the top 30 between differential metabolites and gut microbiota at the genus level. ^*^ *P* < 0.05, ^**^ *P* < 0.01, ^***^ *P* < 0.001.

To further understand the correlation between differential metabolites and microbiota at the genus level, Spearman’s correlation analysis was performed (**Figure 7**). The results showed that the differential metabolites correlated with changes in the microbiota. We found that L-Glutamine is positively correlated with Escherichia-Shigella and Bifidobacterium and negatively correlated with Colidextribacter and Alistipes. Branched-chain amino acids (Leucine and Valine) were positively correlated with Alistipes, Desulfovibrionaceae, Staphylococcus, Blautia, and Muribaculaceae and negatively correlated with Faecalibaculum.

## 4 Discussion

It is increasingly evident that bariatric/metabolic surgery involves multiple weight-independent mechanisms to improve glucose homeostasis and enhance insulin sensitivity and secretion, particularly during specific surgeries (19). In this study, despite observing no significant disparities in body weight between the DJB and SHAM groups, the mice had a relatively lower weight post-DJB surgery. The results of random blood glucose and OGTT showed that DJB significantly improved glucose metabolism and tolerance in obese T2D mice, coupled with a noteworthy increase in serum GLP-1 expression. In addition to stimulating insulin secretion, GLP-1 also promotes insulin biosynthesis as well as β-cell proliferation and survival (20). These findings are consistent with those of previous studies (21, 22) that DJB surgery could be used as a treatment for T2D.

Our study was designed to assess changes in the gut microbial ecology before and after surgery. Marked changes were evident in the microbial composition of CA limbs and COL limbs, with a pronounced increase in the abundance of the Proteobacteria (class: Gammaproteobacteria, order: Enterobacteriales, family: Enterobacteriaceae, genus: Escherichia-Shigella). Similar results were observed in the fecal microbiota of human patients and rats after bariatric surgery (1, 4, 23). At the genus level, we observed a significant decline in Colidextribacter, Blautia, Lachnospiraceae_NK4A136_group, and norank _f_Desulfovibrionaceae. One study reported (24) that Colidextribacter, Blautia, and the Lachnospiraceae_NK4A136_group are closely related to branched-chain amino acids (BCAAs). As important metabolites of the diet or gut microbiome, BCAAs are prevalent and significantly elevated in obese and/or T2D hosts, suggesting that BCAAs can inhibit the function of beta cells in regulating insulin secretion, and are strongly associated with insulin resistance and the risk of developing T2DM (25, 26). Similar results were observed in our study, where BCAA levels decreased significantly in the serum and were associated with Blautia after DJB surgery. This suggests that Alistipes, Desulfovisbrionaceae, Staphylococcus, and Muribaculaceae are closely related to the occurrence and development of T2DM.

Additionally, in BP limbs, A limbs, and C limbs, significant enrichment of Erysipelotrichales (family: Erysipelotrichaceae, genus: Faecalibaculum) after DJB surgery was observed. DJB surgery also resulted in a significant increase in Faecalibaculum and Bifidobacterium and a decrease in Lactobacillus. Increased levels of Bifidobacterium are associated with improved glucose tolerance, glucose-induced insulin secretion, decreased body weight, and decreased levels of inflammatory mediators (27, 28). As previously reported, Faecalibaculum has anti-inflammatory properties and significantly reduces the percentage of intestinal microbiota in patients with T2D (29, 30). Furthermore, SCFAs can inhibit bacterial invasion, maintain intestinal barrier integrity, and regulate inflammatory responses (31). Bifidobacterium and Faecalibaculum are important commensal bacteria that produce SCFAs (22). Lactobacillus, classified as a probiotic, exerts beneficial effects on human health. However, our study revealed a reduction in the abundance of Lactobacilli following DJB surgery. Despite their known benefits, the results of meta-analyses indicate that Lactobacillus may also affect weight gain (32). The number of lactobacilli in obese and obese patients with type 2 diabetes is higher than that in healthy individuals (33, 34), which can be considered in the context of the results of this study. This suggests that the different effects of Lactobacillus on weight may be species- and strain-specific. These studies suggest that DJB surgery not only reduces weight gain but also decreases the number of proximal gut Lactobacillus species present.

Given the proximal intestinal rearrangement after DJB surgery, we cannot overlook the potential impact of microbial changes on the gut barrier and insulin resistance. The results indicated a significant decrease in the expression of the three inflammatory factors in A and BP limbs with a similar trend observed in the C limb. As documented in previous studies, chronic inflammation facilitates the development of T2DM by promoting insulin resistance and β-cell failure while decreasing insulin sensitivity (35–37). Targeted regulation of the inflammatory system can significantly improve hyperglycemia and may gradually become a therapeutic strategy for T2DM (38). Chronic inflammatory diseases of the intestine may lead to the dysregulation of TJ proteins (39). Based on these findings, we investigated the distribution and expression of ZO-1 and claudin-5 in three intestinal segments. DJB altered the distribution and expression of ZO-1 and claudin-5 in the A and C limbs. Notably, both TJ proteins exhibited similar patterns of redistribution after surgery. These results are consistent with those of previous studies, showing that TJ proteins are redistributed under various pathological conditions (40). Our data showed that intestinal permeability decreased in limbs A and C after DJB, whereas there was no significant improvement in the limbs with BP. This is consistent with the results of a study by Yang et al. in rats (41). Additionally, DJB surgery promoted epithelial cell proliferation and adaptive changes in the gut, as evidenced by increased V/C ratios in limbs A and C. These results are consistent with those reported by Li et al. (42). In accordance with previous reports (18), macronutrients, including carbohydrates, proteins, and fats, stimulate intestinal adaptation, which supports the finding that the intestinal permeability of BP limbs does not improve after DJB. In conclusion, strengthening epithelial barrier function and tight junctions may be an adaptive mechanism after DJB.

KEGG analysis revealed that differential metabolites further revealed changes in several pathways. These include membrane transport, amino acid metabolism, and digestive systems, as well as pathways associated with ABC transporters and purine metabolism. Studies have found that ABC transporters (43) play an important physiological role in higher plants and animals and use the energy generated by the hydrolysis of adenosine triphosphate (ATP) to transport various molecules bound to it across the membrane, thereby promoting intestinal cell development. Our KEGG pathway analysis showed that purine metabolism was enriched, suggesting an active microbial reproduction at this stage. Furthermore, the surgery may facilitate transport across the membrane and amino acid digestion and absorption to protect the gut by preventing intestinal atrophy and other metabolic complications. Therefore, we speculate that the surgery may ameliorate diabetes through these pathways. Simultaneously, some metabolites associated with improved glucose metabolism increased after the DJB surgery. Glutamine levels are significantly elevated after DJB surgery and are involved in multiple metabolic pathways. Glutamine is the main energy source for intestinal cells and can regulate the phosphorylation of tight junction proteins, thereby improving the intestinal barrier function (44). Glutamine supplementation may maintain intestinal homeostasis through intestinal microbial metabolites, improve intestinal immunity, and alleviate intestinal inflammation (45). Moreover, studies have shown that bile acids play important roles in glucose homeostasis (46). Changes in gastrointestinal anatomy may also affect the enterohepatic recirculation of bile acids, possibly mediating an increase in GLP-1 through increased uptake of bile acids in the intestine, helping to improve blood glucose (47).

However, this study has some limitations. Firstly, in the analysis of gut microbial diversity, there was no statistically significant difference between the groups. Since we studied proximal gut microbes instead of fecal microbes, this may indicate a substantial similarity in the proximal gut microbiota. In addition, similar body weights may be a possible mechanism for the undifferentiated gut microbiome. Second, because the sample size was small and there was a lack of negative control groups for the gut microbiome analysis, the correlation was not verified. In future studies, it will be necessary to expand the sample size and conduct further validation. Finally, the current study mainly focused on the effect of intestinal rearrangement in the proximal small intestine on improving glucose metabolism after DJB surgery, and did not explore the specific effect on blood lipids. In addition, this study only explored the effects of proximal intestinal structural rearrangements on gut microbiota and metabolism, and further studies are needed to explore the underlying mechanisms.

In summary, we provide a valuable mouse model for investigating the mechanism by which T2D improves without weight loss and provide new insights into the BP, A, and C limbs after DJB surgery. Our results reveal that the post-DJB changes in the gut microbiota and metabolites were sufficiently robust to attenuate inflammatory responses and intestinal permeability, consequently bolstering the stability of glycemic metabolism. These findings suggest that proximal intestinal rearrangements play an important role in surgery-related improvements in patients with T2DM.

## Data Availability

The datasets presented in this study can be found in online repositories. The names of the repository/repositories and accession number(s) can be found below: https://www.ncbi.nlm.nih.gov/, PRJNA1087303.
