# Supplementary material for: Intestinal rearrangement of biliopancreatic limbs, alimentary limbs, and common limbs in obese type 2 diabetic mice after duodenal jejunal bypass surgery
Source: Front Endocrinol (Lausanne). 2025 Jan 8;15:1456885. doi: 10.3389/fendo.2024.1456885 (PMC11750664; doi:10.3389/fendo.2024.1456885)
Supplement: Supplementary file 1 [file DataSheet1.docx]

**Supplementary Table 1**

Primer sequences for quantitative real-time polymerase chain reaction.

| **Gene (Mouse)** | **Forward primer** | **Reverse primer** |
| --- | --- | --- |
| *Tjp1* | TCTCTGTTCACACAGATAAGCCT | TCTTCCACAGCTGAAGGACTC |
| *Cldn5* | TAAGGCACGGGTAGCACTCA | GGACAACGATGTTGGCGAAC |

**Supplementary Table 2**

At the genus level, statistically significant differences in relative abundance of gut

microbiota in the CA and COL limbs between the SHAM group and the DJB group (8 weeks post-surgery). Values were expressed as mean ± SD.

|  | **SHAM Gruop** | **DJB Gruop** | **P_value** |
| --- | --- | --- | --- |
| **Caeum limb** |  |  |  |
| g__norank_f__Desulfovibrionaceae | 12.72 ± 3.221 | 0.6952 ± 0.3129 | 0.003 |
| g__Romboutsia | 0.1111 ± 0.05535 | 0.006892 ± 0.008829 | 0.03227 |
| g__Enterobacter | 0 ± 0 | 0.02044 ± 0.01236 | 0.0457 |
| g__Corynebacterium | 0.001933 ± 0.00005728 | 0 ± 0 | 5.12E-07 |
| **Colon limb** |  |  |  |
| g__Colidextribacter | 8.787 ± 0.9059 | 0.8201 ± 0.5317 | 0.000194 |
| g__Blautia | 7.389 ± 2.39 | 1.926 ± 0.7399 | 0.01941 |
| g__Lachnospiraceae_NK4A136_group | 2.907 ± 1.071 | 0.6248 ± 0.4628 | 0.0276 |
| g__Oscillibacter | 1.135 ± 0.5415 | 0.1575 ± 0.02126 | 0.03537 |
| g__unclassified_f__Oscillospiraceae | 0.9508 ± 0.3033 | 0.1081 ± 0.05142 | 0.009004 |
| g__Tuzzerella | 0.5864 ± 0.2071 | 0.1132 ± 0.1028 | 0.02389 |
| g__GCA-900066575 | 0.4501 ± 0.1634 | 0.06553 ± 0.04015 | 0.0167 |
| g__unclassified_f__Ruminococcaceae | 0.2956 ± 0.1093 | 0.07661 ± 0.04885 | 0.0339 |
| g__Lachnospiraceae_UCG-006 | 0.1955 ± 0.07562 | 0.009275 ± 0.002771 | 0.01303 |
| g__Streptococcus | 0.1269 ± 0.06226 | 0.0115 ± 0.01734 | 0.0365 |
| g__Lachnospiraceae_FCS020_group | 0.04507 ± 0.01574 | 0.00438 ± 0.002006 | 0.01131 |
| g__Parvibacter | 0.01036 ± 0.00568 | 0.02611 ± 0.005958 | 0.02952 |
| g__norank_f__Christensenellaceae | 0.01949 ± 0.007508 | 0.002845 ± 0.003081 | 0.02374 |

**Supplementary Table 3**

At the genus level, statistically significant differences in relative abundance of gut microbiota in the BP, A and C limbs between the SHAM group and the DJB group (8 weeks post-surgery). Values were expressed as mean ± SD.

|  | SHAM Group | DJB Group | P_value |
| --- | --- | --- | --- |
| Biliopancreatic limb |  |  |  |
| g__Faecalibaculum | 8.069 ± 3.753 | 23.04 ± 7.373 | 0.03502 |
| g__Streptococcus | 1.011 ± 0.3156 | 0.252 ± 0.2301 | 0.02815 |
| g__Staphylococcus | 0.3885 ± 0.1978 | 0.01234 ± 0.01016 | 0.03023 |
| g__Desulfovibrio | 0.001726 ± 0.002989 | 0.1012 ± 0.06047 | 0.04658 |
| g__Lachnospiraceae_NK4A136_group | 0.04101 ± 0.01898 | 0.003516 ± 0.001569 | 0.02702 |
| g__Burkholderia-Caballeronia-Paraburkholderia | 0.02862 ± 0.009866 | 0 ± 0 | 0.007363 |
| g__Pseudogracilibacillus | 0.01169 ± 0.006153 | 0.0008733 ± 0.001513 | 0.0417 |
| g__Tepidimicrobium | 0.005304 ± 0.001468 | 0.0008664 ± 0.001501 | 0.02155 |
| Alimentary limb | | | |
| g__Bifidobacterium | 0.06355 ± 0.08 | 0.5158 ± 0.1392 | 0.008165 |
| g__Gallicola | 0.02616 ± 0.01023 | 0.005708 ± 0.003087 | 0.02954 |
| g__Tepidimicrobium | 0.001613 ± 0.002793 | 0.01042 ± 0.002384 | 0.01422 |
| g__Jeotgalibaca | 0.0109 ± 0.00601 | 0 ± 0 | 0.03481 |
| g__Globicatella | 0.002847 ± 0.0004834 | 0 ± 0 | 0.0005205 |
| Common limb | | | |
| g__Lactobacillus | 32.36 ± 6.958 | 13.58 ± 9.309 | 0.04886 |
| g__Bifidobacterium | 0.005297 ± 0.007319 | 1.014 ± 0.4788 | 0.02181 |
| g__Clostridium_sensu_stricto_1 | 0.03333 ± 0.04046 | 0.6424 ± 0.355 | 0.04189 |
| g__Bacillus | 0.0334 ± 0.01719 | 0.004715 ± 0.004624 | 0.04926 |
| g__Sinibacillus | 0.01752 ± 0.003434 | 0.005934 ± 0.005267 | 0.03314 |
| g__norank_f__norank_o__Clostridia_UCG-014 | 0.01303 ± 0.006021 | 0.001835 ± 0.001698 | 0.03619 |
| g__norank_f__Ruminococcaceae | 0.01145 ± 0.002481 | 0.0007185 ± 0.001244 | 0.002583 |
| g__Tepidimicrobium | 0.009156 ± 0.002328 | 0.0005585 ± 0.0009673 | 0.004113 |

**Supplementary Table 4**

107 different serum metabolites between DJB group and SHAM group (8 weeks after surgery).

| **Metabolite** | **Regulate** | **Mode** | **FC(DJB/SHAM)** | **P_value** | **FDR** |
| --- | --- | --- | --- | --- | --- |
| Tauro-alpha-muricholic acid | up | pos | 1.1476 | 0.00745 | 0.1041 |
| N-(1-Deoxy-1-fructosyl)leucine | down | pos | 0.9267 | 0.009699 | 0.1192 |
| N-(1-Deoxy-1-fructosyl)valine | down | pos | 0.9198 | 0.01525 | 0.1424 |
| Citrulline | up | pos | 1.0488 | 0.001232 | 0.05313 |
| (5Z,8Z,10E,14Z)-12-oxoicosa-5,8,10,14-tetraenoic acid | down | pos | 0.8556 | 0.01545 | 0.1424 |
| D-Pipecolic acid | up | pos | 1.0806 | 0.000784 | 0.04486 |
| 5-L-Glutamyl-L-alanine | up | pos | 1.0853 | 0.003681 | 0.07439 |
| THTC | up | pos | 1.0631 | 0.01562 | 0.1428 |
| Ethyl glucuronide | up | pos | 1.0928 | 0.01489 | 0.1418 |
| Oxidized glutathione | up | pos | 1.0539 | 0.01619 | 0.1447 |
| N-(1-Deoxy-1-fructosyl)phenylalanine | down | pos | 0.9521 | 0.01631 | 0.145 |
| Benzaldehyde | up | pos | 1.0442 | 0.001188 | 0.05274 |
| Pantothenic Acid | up | pos | 1.0375 | 0.01055 | 0.1231 |
| 2-(2-oxo-1,3-dihydroindol-3-yl)acetic acid | up | pos | 1.05 | 0.009739 | 0.1192 |
| 1-(1,2,3,4,5-Pentahydroxypent-1-yl)-1,2,3,4-tetrahydro-beta-carboline-3-carboxylate | down | pos | 0.9262 | 0.00641 | 0.09693 |
| Trans-3-Indoleacrylic acid | up | pos | 1.023 | 0.02766 | 0.1745 |
| Choline Glycerophosphate | down | pos | 0.9545 | 0.0007021 | 0.04218 |
| Chol-11-Enic Acid | up | pos | 1.1253 | 0.04009 | 0.2065 |
| 6-[7,8-dihydroxy-3,5-dioxo-9-(sulfooxy)-1H,2H,3H,5H-cyclopenta[c]isochromene-1-carbonyloxy]-3,4,5-trihydroxyoxane-2-carboxylic acid | up | pos | 1.0834 | 0.001476 | 0.05313 |
| L-arginine | up | pos | 1.0516 | 0.00378 | 0.07465 |
| L-Proline | up | pos | 1.0591 | 0.004684 | 0.08359 |
| Uric acid | down | pos | 0.9538 | 0.002558 | 0.06255 |
| Dimethyl malonic acid | up | pos | 1.0687 | 0.02313 | 0.1645 |
| Glutamylphenylalanine | up | pos | 1.0547 | 0.04403 | 0.2121 |
| Alanylalanine | up | pos | 1.0849 | 0.002729 | 0.065 |
| 2'-O-Methyl-5-methylcytidine | down | pos | 0.8411 | 0.006135 | 0.09536 |
| (±)-Propionylcarnitine | up | pos | 1.0787 | 0.003065 | 0.06821 |
| Uracil | up | pos | 1.0325 | 0.02841 | 0.176 |
| Guanosine | up | pos | 1.5278 | 1.41E-05 | 0.004994 |
| Xanthosine | up | pos | 1.0469 | 0.01259 | 0.1323 |
| Gamma-Glutamylvaline | up | pos | 1.047 | 0.04847 | 0.219 |
| Butyryl-L-carnitine | up | pos | 1.0829 | 0.02677 | 0.174 |
| N-Cinnamoylglycine | up | pos | 1.0357 | 0.02623 | 0.172 |
| 4-formyl Indole | up | pos | 1.0287 | 0.0334 | 0.1888 |
| Apocholic acid | up | pos | 1.3251 | 0.004649 | 0.0835 |
| (5Z,8Z,11Z,13E)-15-oxoicosa-5,8,11,13-tetraenoic acid | down | pos | 0.9487 | 0.02174 | 0.1616 |
| (24R,25R)-25,26-epoxy-1alpha,24-dihydroxyvitamin D3 | up | pos | 1.1613 | 0.0009947 | 0.04658 |
| LysoPC(22:6(4Z,7Z,10Z,13Z,16Z,19Z)) | down | pos | 0.9442 | 0.007214 | 0.1021 |
| 7-Dehydrocholesterol 5,6-oxide | up | pos | 1.1223 | 0.003305 | 0.06922 |
| Sphingosine-1-phosphate | up | pos | 1.0244 | 0.007706 | 0.1055 |
| Taurocholate | up | pos | 1.1055 | 0.01734 | 0.1486 |
| Laxogenin | up | pos | 1.2477 | 0.0002877 | 0.02706 |
| (4E,7E,10Z,13E,16E,19E)-docosa-4,7,10,13,16,19-hexaenoic acid | down | pos | 0.9345 | 0.0007532 | 0.04354 |
| Linoleamide | up | pos | 1.0294 | 0.04568 | 0.2136 |
| Glutamine-betaxanthin | down | pos | 0.9313 | 0.006907 | 0.1009 |
| N-Propionylmethionine | up | pos | 1.0489 | 0.007346 | 0.1032 |
| 2-Methylbutyroylcarnitine | up | pos | 1.0562 | 0.0005532 | 0.03936 |
| Nicotyrine | up | pos | 1.0316 | 0.02396 | 0.1656 |
| D-beta-Homophenylalanine | up | pos | 1.0309 | 0.03422 | 0.1908 |
| 5'-Deoxy-5'-(methylthio)adenosine | up | pos | 1.1225 | 0.0001328 | 0.0222 |
| Thymine | up | pos | 1.0915 | 0.005319 | 0.08865 |
| Toluene | up | pos | 1.0363 | 0.001952 | 0.05842 |
| Gamma-Glutamylmethionine | up | pos | 1.148 | 0.04444 | 0.2121 |
| (?)-Norepinephrine | down | pos | 0.9697 | 0.03548 | 0.193 |
| Sh373-1 | down | pos | 0.9141 | 0.03782 | 0.1999 |
| N-Methyl-4-pyridone-3-carboxamide | down | pos | 0.9418 | 0.004166 | 0.07919 |
| Guanine | up | pos | 1.4664 | 0.0002003 | 0.02392 |
| 3-Acetyl-5-sec-butyl-4-hydroxy-1,5-dihydro-2H-pyrrol-2-one | down | pos | 0.8973 | 0.007728 | 0.1055 |
| (R)-3-hydroxybutyrylcarnitine | up | pos | 1.0423 | 0.01941 | 0.1557 |
| Phenylacetaldehyde | up | pos | 1.0278 | 0.03965 | 0.2058 |
| Acetylcholine | up | pos | 1.0485 | 0.002143 | 0.05893 |
| Betaine | up | pos | 1.0399 | 0.02209 | 0.1616 |
| DL-Homoserine | up | pos | 1.0525 | 0.01782 | 0.1503 |
| 4-Pyrimidine Methanamine (hydrochloride) | up | pos | 1.0602 | 0.01782 | 0.1503 |
| Chrycorin | down | pos | 0.92 | 0.0002752 | 0.02706 |
| Indole | up | pos | 1.0397 | 0.001345 | 0.05313 |
| Phenol | up | pos | 1.0367 | 0.001465 | 0.05313 |
| N-Acetyltaurine | down | neg | 0.9648 | 0.001818 | 0.03768 |
| L-Erythrulose | down | neg | 0.9229 | 0.0001035 | 0.01454 |
| Dihydro-5-(2-octenyl)-2(3H)-furanone | up | neg | 1.0423 | 0.02951 | 0.1316 |
| Xanthine | up | neg | 1.0979 | 0.0031 | 0.0461 |
| AMINOHYDROXYBUTYRIC ACID | up | neg | 1.077 | 0.002616 | 0.04238 |
| Glutaric anhydride | down | neg | 0.9425 | 4.81E-05 | 0.01426 |
| Uridine | up | neg | 1.1102 | 0.001581 | 0.03531 |
| Inosine | up | neg | 1.2548 | 0.001304 | 0.03457 |
| Phenylacetylglycine | up | neg | 1.0736 | 0.02287 | 0.1149 |
| [3-(4-methoxyphenyl)propoxy]sulfonic acid | up | neg | 1.1278 | 0.04631 | 0.1589 |
| 3-Dehydrocholic acid | up | neg | 1.3039 | 0.04343 | 0.1553 |
| Deoxycholic acid | up | neg | 1.0493 | 0.04766 | 0.1622 |
| Hecogenin | up | neg | 1.1455 | 0.0001618 | 0.01806 |
| (6E,10E)-3,7,11,15-tetramethylhexadeca-1,6,10,14-tetraene-3,5,9-triol | down | neg | 0.959 | 0.01453 | 0.09466 |
| 4,5-dehydro Docosahexaenoic Acid | down | neg | 0.9126 | 0.003655 | 0.04919 |
| DG(15:0/20:2(11Z,14Z)/0:0) | up | neg | 1.0298 | 0.01831 | 0.1032 |
| 1-Linoleoylglycerol | down | neg | 0.8161 | 0.02852 | 0.13 |
| Mucronine D | down | neg | 0.8853 | 0.04869 | 0.1636 |
| Stearoyllactic acid | down | neg | 0.9626 | 0.03794 | 0.1456 |
| DG(18:0/0:0/18:2n6) | down | neg | 0.9681 | 0.04404 | 0.1557 |
| 6,8-Tricosanedione | up | neg | 1.0547 | 0.04181 | 0.1533 |
| (+)-18-methyl-eicosanoic acid | down | neg | 0.7145 | 0.01416 | 0.09432 |
| Phosphatidylethanolamine lyso 20:5 | up | neg | 1.0589 | 0.001279 | 0.03457 |
| 2-Hydroxymyristic Acid | down | neg | 0.9229 | 0.006186 | 0.06265 |
| 4-HDoHE | down | neg | 0.9504 | 0.0006126 | 0.02666 |
| Tauroursodeoxycholic acid | up | neg | 1.1191 | 0.01844 | 0.1032 |
| 5(S)-HEPE | down | neg | 0.9344 | 0.003654 | 0.04919 |
| Ponasterone | up | neg | 1.1754 | 0.0003137 | 0.02192 |
| 12S-HHTrE | up | neg | 1.0486 | 0.02313 | 0.1155 |
| (9Z,12E)-15,16-dihydroxyoctadeca-9,12-dienoic acid | up | neg | 1.0726 | 0.04915 | 0.1643 |
| S-Lactoylglutathione | down | neg | 0.9272 | 0.004854 | 0.05582 |
| L-Phenylalanine | up | neg | 1.0425 | 8.97E-05 | 0.01454 |
| L-Histidine | up | neg | 1.0995 | 0.001349 | 0.03457 |
| Myo-Inositol | down | neg | 0.9702 | 0.0001541 | 0.01806 |
| L-Glutamine | up | neg | 1.0367 | 0.007438 | 0.06814 |
| Taurine | up | neg | 1.0412 | 0.002737 | 0.04306 |
| (3R,4S,5R)-5-(hydroxymethyl)oxolane-2,3,4-triol | up | neg | 1.0341 | 0.03999 | 0.1496 |
| D-Glyceric acid | up | neg | 1.0848 | 0.04281 | 0.1544 |
| D-Tartaric acid | up | neg | 1.1383 | 0.01674 | 0.09984 |
| Thiosulfate | up | neg | 1.0443 | 0.0322 | 0.1372 |

**Supplementary Table 5**

Glutamine is involved in different metabolic pathways to improve glucose homeostasis.

|  | Metab ID | Retention time | KEGG Compound ID | Mode |
| --- | --- | --- | --- | --- |
| ABC transporters | | | | |
| L-arginine | metab_820 | 0.8636 | C00062 | pos |
| L-Proline | metab_900 | 1.042 | C00148 | pos |
| Guanosine | metab_1475 | 2.7938 | C00387 | pos |
| Xanthosine | metab_1515 | 2.9242 | C01762 | pos |
| Betaine | metab_5629 | 0.9593 | C00719 | pos |
| Uridine | metab_6577 | 2.5118 | C00299 | neg |
| Inosine | metab_6595 | 3.0041 | C00294 | neg |
| L-Phenylalanine | metab_8581 | 3.4556 | C00079 | neg |
| L-Histidine | metab_8754 | 1.1031 | C00135 | neg |
| Myo-Inositol | metab_8819 | 0.9507 | C00137 | neg |
| L-Glutamine | metab_8823 | 0.9469 | C00064 | neg |
| Taurine | metab_8904 | 0.806 | C00245 | neg |
| (3R,4S,5R)-5-(hydroxymethyl)oxolane-2,3,4-triol | metab_8905 | 0.8023 | C00121 | neg |
| Thiosulfate | metab_8965 | 0.7676 | C00320 | neg |
| Purine metabolism |  |  |  |  |
| Uric acid | metab_1235 | 2.0487 | C00366 | pos |
| Guanosine | metab_1475 | 2.7938 | C00387 | pos |
| Xanthosine | metab_1515 | 2.9242 | C01762 | pos |
| Guanine | metab_4961 | 2.7938 | C00242 | pos |
| Xanthine | metab_6290 | 1.1595 | C00385 | neg |
| Inosine | metab_6595 | 3.0041 | C00294 | neg |
| L-Glutamine | metab_8823 | 0.9469 | C00064 | neg |
| Protein digestion an absorption |  |  |  |  |
| L-arginine | metab_820 | 0.8636 | C00062 | pos |
| L-Proline | metab_900 | 1.042 | C00148 | pos |
| Indole | metab_6173 | 3.0727 | C00463 | pos |
| Phenol | metab_6174 | 3.0798 | C00146 | pos |
| L-Phenylalanine | metab_8581 | 3.4556 | C00079 | neg |
| L-Histidine | metab_8754 | 1.1031 | C00135 | neg |
| L-Glutamine | metab_8823 | 0.9469 | C00064 | neg |

**Supplementary Table 6**

Statistically significant differences in relative abundance of short-chain fatty acids between the DJB group and the SHAM group (8 weeks post-surgery).

| **Relative abundance (%)** | **SHAM Group** | **DJB Group** | **P_value** |
| --- | --- | --- | --- |
| 2-(2-oxo-1,3-dihydroindol-3-yl)acetic acid | 5.401 ± 0.08996 | 5.671 ± 0.04569 | 0.009739 |
| Dimethyl malonic acid | 5.171 ± 0.157 | 5.526 ± 0.07059 | 0.02313 |
| Amino hydroxybutyric acid | 5.246 ± 0.1041 | 5.65 ± 0.01133 | 0.002616 |

**Supplementary Material**

**Surgical procedure:**

**1 General preoperative preparation**

1.1 Fast the mice for 8 hours before the surgery. Withhold water 2 hours before the surgery.

1.2 Administer 1% sodium pentobarbital solution (6 mL/kg) and buprenorphine (1 mg/kg) intraperitoneally. Securely clamp the toes or tails of the mice with forceps, ensuring no visible signs of twitching or shaking are observed. Under appropriate anesthesia, the mice can breathe freely without requiring supplemental oxygen.

1.3 Position the mice supine on a sterile board under a stereomicroscope while applying eye ointment to their eyes for protection. Maintain warmth throughout the procedure using an electric blanket specifically designed for rodents' comfort and safety measures such as surgical gowns, sterile gloves, and autoclaved instruments should be strictly followed.

**2 Duodenal jejunal bypass: Surgical Procedure**

2.1 The stomach along with the pylorus lies beneath the liver while the ligament of Treitz is located at the distal duodenum. Jejunum situated distally by approximately 5 cm from the ligament of Treitz was double-ligated using a silk suture. Cut the jejunum at the midpoint of the two ligations and suture the jejunal stump with a 10-0 silk suture.

2.2 Pull the proximal jejunal incision 5 cm along the bowel to the jejunum to create a jejunal-jejunal anastomosis. Align the two bowels horizontally, and then use a 10-0 silk suture to create the lateral anastomosis.

2.3 Secure both ends of the bowel and cut the incision to equal length. Suture the second layer of the posterior intestinal wall with a full-thickness continuous suture.

2.4 Secure both ends of the bowel and suture the anterior wall of the bowel. Suture the first layer of the anterior wall with a simple continuous suture and suture the second layer with a horizontal varus suture.

2.5 Pull the distal jejunal incision into the duodenum 1 cm below the pylorus to create a duodenal-jejunal anastomosis. Suture it in the same method as in steps 2.4-2.6.

2.6 Ligate the bowel with the micro forceps and cut the bowel with the micro scissors. Double ligate the duodenum with a 6-0 silk suture 2 mm from the distal end of the duodenal-jejunal anastomosis, cut at the midpoint with micro scissors, and suture the stump with a 10-0 silk suture.

2.7 Rinse the abdominal cavity with 30 ℃ saline. Return the bowel to the abdominal cavity. Suture the muscle and the skin separately with a 6-0 silk. Then disinfect the skin with iodophor.

2.8 After surgery, inject 30 ℃ saline (30 mL/kg) subcutaneously in the back to prevent dehydration. Inject penicillin (10 mg/kg) intramuscularly to prevent infection.

**3. General postoperative care**

3.1 After surgery, place the mice on an electric blanket to prevent hypothermia. Allow the mice to crawl freely until they are fully awake before returning to their cages.

3.2 On the postoperative day, restrict the food and water, and inject 2 mL of saline subcutaneously into the back of the mice. On the first postoperative day, give 10 mL of 10% glucose and a functional drink (1:1 ratio) without food, and inject 1 mL of saline subcutaneously.

3.3 On the second and third postoperative days, feed the mice with a mixture of 20 ml of 10% glucose and a functional drink. After the fourth day, give the mice pure water and a high-fat diet. Transitional feeding should be performed according to the postoperative recovery status in the following order: solution, semi-liquid, or solid food.

3.4 Postoperative analgesia: inject buprenorphine (0.1 mg/kg) every 12 hours from days 1 to 3, and then once daily until day 5. After surgery, observe the feeding conditions, activity, feces, and wound healing of the mice.
